# Distinguishing social and medical traits: perspectives of scientists using polygenic scores

**DOI:** 10.1007/s12687-026-00857-z

**Published:** 2026-02-14

**Authors:** Margaret Waltz, Karen M. Meagher, Courtney Canter, Matthew Kucmanic, Kristine J. Kuczynski, Anya E. R. Prince, R. Jean Cadigan

**Affiliations:** 1 Department of Social Medicine, University of North Carolina at Chapel Hill, Chapel Hill, NC USA; 2 Center for Bioethics, University of North Carolina at Chapel Hill, Chapel Hill, NC USA; 3 Center for Health Justice and Bioethics, Temple University Lewis Katz School of Medicine, Philadelphia, PA USA; 4 Department of Anthropology, University of North Carolina at Chapel Hill, Chapel Hill, NC USA; 5 School of Earth, Environment, and Sustainability, University of Iowa, Iowa City, IA USA; 6 College of Law, University of Iowa, 130 Byington Road, Iowa City, IA 52242 USA

**Keywords:** Polygenic scores, Scientist interviews, Social traits, Medical traits, ELSI

## Abstract

Polygenic scores (PGS) have been developed for a wide variety of traits, such as cancer, risk tolerance, obesity, asthma, educational attainment, and cardiovascular disease. Prior research shows that the public tends to view the use of genetic information for medical traits more favorably than for social or other non-medical traits, and legal and policy discourse regularly treats medical and social traits as distinct categories. However, distinguishing between social and medical traits can be conceptually and practically challenging. Drawing on 47 semi-structured interviews with researchers who have developed or utilized PGS across a range of traits, this study examines how scientists discussed and conceptualized a dividing line between what is medical and what is social, as well as the perceived necessity of a medical/social divide. Overall, the scientists we interviewed broadly agreed that the line is ambiguous; they did not hold unequivocal views about what constitutes a social versus a medical trait, nor where a clear line might be drawn. Instead, they reasoned through any distinctions between social and medical traits in varied and sometimes conflicting ways, including attempting to draw the line, highlighting the role of social context in drawing the line, questioning the line’s relevance, and pointing to the line’s policy implications. We argue that this ambiguity has significant implications for how PGS research is conducted, interpreted, and regulated, calling for a reexamination of regulatory strategies that rely on this distinction.

## Introduction

Genome-wide association studies (GWAS) have opened the door to the creation of polygenic scores (PGS), where researchers aggregate influences of genetic variants to correlated phenotypes or traits by leveraging data from large biobanks (Mills and Tropf 2020). PGS can be used to explore the genetic associations with essentially any trait of interest for which there is large scale data (Choi et al. 2020). PGS have thus been created for a wide variety of phenotypes, such as cancer, risk tolerance, obesity, asthma, educational attainment, and cardiovascular disease (Sud et al. 2021; Karlsson Linnér et al. 2019; Jansen et al. 2024; Sordillo et al. 2021; Okbay et al. 2022; Livingstone et al. 2021).

The ethical, legal, and social implications (ELSI) of PGS are increasingly being examined in U.S. health care as genetic research advances beyond testing and disclosure of single-gene variants or traits (Dudbridge 2013; Visscher et al. 2017; eMERGE Consortium 2021). At the same time, researchers in sociogenomics are using PGS to investigate genetic contributions to social and behavioral traits (Braudt 2018; Conley 2016; Mills and Tropf 2020; Harden and Koellinger 2020). Yet social and behavioral traits are sometimes challenging to distinguish from medical traits, as medicalization itself is a social phenomenon (Conrad 2007). For example, personality traits, such as “the big five” (openness to experiences, neuroticism, agreeableness, extraversion, and conscientiousness), can be studied for their contribution to mental health or for their normal distribution and manifestation beyond the health sector (McCrae and John 1992).

While challenging to distinguish social and medical traits, empirical research has shown that the public views the use of genetic information for medical traits more favorably than for social or non-medical traits. Zhang and colleagues (2021) reported higher approval for PGS use for diseases, such as schizophrenia and diabetes, compared to traits like IQ, height, or skin tone. Similarly, Furrer and colleagues (2024) found greater public support for polygenic embryo screening for conditions like cancer, heart disease, and Alzheimer’s disease than for traits like intelligence or skin color. Interviews with clinicians and patients by Barlevy and colleagues (2024) echoed this pattern, though they noted that distinctions between physical or psychiatric health conditions, like diabetes or depression, and physical and cognitive traits, like height or intelligence, were often blurred.

This pattern of favoring medical over social traits extends beyond polygenic scores to other genetic technologies, such as gene editing. Studies have shown that the public, scientists, and clinicians are generally more supportive of gene editing when used to treat or prevent health conditions or diseases, such as muscular dystrophy or cystic fibrosis, than when applied to modify or potentially enhance genes associated with traits like intelligence, appearance, personality, or even traits that straddle medical and social domains, like Type 2 diabetes and obesity (Scheufele et al. 2017; Pew Research Center 2018; Waltz et al. 2021; Armsby et al. 2019; Riggan et al. 2019). Similarly, legal and policy discourse regularly distinguishes between medical and social genetic testing, treating them as distinct categories (Callier and Prince 2024; Kasperbauer and Wright 2020; Spector-Bagdady et al. 2018).

This continued distinction raises questions of whether and where to draw the line between medical and social traits. Drawing on interviews with researchers who have developed or utilized PGS across a range of traits, we report on how interviewees discussed and conceptualized a dividing line between what is medical and what is social, as well as the perceived necessity of the medical/social divide.

## Methods

We identified potential interview participants through a literature search of articles related to the development or use of polygenic scores using a range of databases, such as HeinOnline, Google Scholar, and the preprint databases bioRxiv and medRxiv. To capture a range of what might be considered social or medical, we purposefully selected PGS articles based on diverse traits identified across the scientific and medical literature such as cardiovascular disease and educational attainment (Kumuthini et al. 2022; Canter et al. 2025). We recorded the first and last authors of these 337 articles and identified their contact information. After removing duplicate names and excluding those for whom we could not locate current contact information, we identified 296 unique authors. Using purposive sampling, we then prioritized authors based on the specific traits of interest relevant to our study and emailed 187 potential participants with an explanation of the goals of our study. Participants provided verbal consent prior to beginning the interview. The University of North Carolina at Chapel Hill Institutional Review Board deemed the study to be exempt from federal human subjects research regulations.

Between October 2024 and May 2025, three social scientists on the team (MW, CC, RJC) conducted 47 semi-structured interviews with researchers who had developed or used polygenic scores in their work, a 25% response rate. Interviews covered a range of topics, including perceptions of the potential benefits and harms of using PGS within or outside of research, the limitations of PGS, ideal measures of traits for the development of PGS, and distinctions between medical and social traits, the last domain being the focus of the present analysis. Participants were asked: “What thoughts do you have on how to distinguish a medical trait from a social trait?” and “How important do you think it is for researchers to make this distinction in their work?” Given the semi-structured interview format, interviewers also used follow-up questions and probes to explore participants’ examples, elicit examples, clarify reasoning, and pursue emerging lines of discussion. Interviews were held via Zoom, lasted approximately 60 minutes, and were audio-recorded with participant consent. Audio-recordings were then professionally transcribed verbatim for analysis. Interviewers wrote field notes after each interview, noting their impressions and highlighting notable discussions or questions raised by participants.

To analyze the interviews, team members (MW, CC, MK) conducted qualitative analytic memoing, which involves synthesizing responses to research questions, interpretating patterns, identifying emergent themes and connections across interviews, and selecting illustrative quotes that support these findings (Patel et al. 2016; Groenewald 2008). We developed a preliminary memoing template based on the interview guide, and the memoing team met regularly to discuss potential emerging themes, which were then added to the template. The template was piloted using a subset of interviews to ensure the breadth of relevant topics were captured, and team members then worked in pairs to ensure they were filling out the memo templates consistently. Once consistency was reached, team members memoed individually, attending regular meetings to resolve any questions as memoing proceeded.

The present analysis stems from the “Social vs. Medical” memo category, which captured any discussions throughout the interview of how interviewees draw a line between social and medical traits; how important interviewees think it is for researchers to make distinctions between social and medical, and why; how interviewees discussed or categorized traits as medical or social; and how interviewees referenced or discussed medicalization. The lead author then conducted a secondary review of the transcripts to confirm the contextual accuracy of previously identified quotes and identify additional illustrative quotes. These quotes, along with patterns noted across memos, were presented to the research team for discussion. This analytic process resulted in the identification of the themes presented below, which reflect patterns of thought that existed within and across interviews.

## Results

Interviewees reasoned through the distinctions between social and medical traits in varied and sometimes conflicting ways, including attempting to draw the line, highlighting the role of context, questioning its relevance, and pointing to its policy implications.

### Attempting to draw the line

Some interviewees explored ways to draw the line between social and medical traits by carving out what constitutes a medical trait. To identify medical traits, one could look at which traits have medical treatments. For instance, one interviewee said of medical traits, “Intuitively, it’s like what would you go to a clinic and get a prescription for” (Participant (P) 1). Another interviewee said, “I don’t know. Whether you have a medication or treatment for that phenotype could be a way. Like for obesity, alcohol-use, we have treatment for that. But life satisfaction, I don’t know whether there’s any way to control that…” (P2). Yet another stated, “I guess medical traits are true medical traits because they can be treated, or we can understand how we can treat them…. I don’t know” (P3). One interviewee offered an example that suggested that along with the possibility of treatment, medical traits are those discussed or managed in clinical encounters: “I do think of obesity as medical. BMI is one of the vital signs we take for all of our patients who come to clinic. And we have now obesity lowering medications, and we have always counseled on diet and exercise. So, I do see that as kind of like mine, mine being like the doctor’s purview” (P4).

Some interviewees reasoned that it was easier to identify medical traits than social traits because there are “specific criteria” or “definitions” for medical traits or syndromes, like bipolar disorder or depression, but there are not clinical definitions for more social traits, like happiness (P5). Others said it is easier to identify a medical trait because of the potential for diagnoses or treatment:

Anything sort of in that range that can be diagnosed by a doctor in some form, or I guess if it’s treated or managed by a health professional in some way, I would say that’s a medical trait. And I don’t think everything else is a social trait. I don’t know, things like height and BMI, they’re not social traits arguably, but they’re also not necessarily medical either… In my head it seems easier to define what a medical trait is and then maybe most other things then fall under a social trait (P6).

Echoing this sentiment, an interviewee said that “a medical trait is something a doctor can provide a medical diagnosis about…” (P7). However, they said they were “not sure if this would be a perfect distinction” and noted the importance of interpretation, which can reflect underlying disagreement about whether a trait is social or medical:

I think if someone, for instance, does research on…a trait that I would feel very medical, I don’t know, schizophrenia, for instance…, I think I would assume that people—readers understand it is a medical trait and not a social trait, but maybe the other way around is more difficult. So for instance…., well-being—I think it would be considered a social trait, but then other people see it as sort of an extension of depression, right, which would make it a more medical trait, so it’s probably important to say something about this, but as we are not sure where this boundary lies, it’s a bit difficult to do this (P7).

### The role of context in drawing the line

Some interviewees challenged the utility of using clinical criteria to label medical traits by reasoning that distinctions between social and medical traits are constructed or shaped by social contexts. One interviewee, for example, said they had “no clue” of where to draw the line and pointed to ADHD, saying:

[T]here’s a medical definition, like diagnostic criteria… But then those criteria have evolved over time immensely. Way more people are fitting in that category now. And we also have this neurodiversity movement, which understands ADHD as kind of a normal part of the spectrum of human differences and focuses more on how schools and workplaces and society in general are not adapted to this full spectrum of differences and shifts the focus on how society can change (P8).

Another argued that the question of how to distinguish social and medical traits “is not really a scientific question but is more like a moral or ethical question” (P9). They similarly commented on “all the neurodivergence traits,” saying “they are the perfect example. Where you’re like, okay is this an actual problem? Is it society that needs to change? Is this like, why is there more now? It is not evolution, so it must be society is changing somehow” (P9). Expanding on the idea of social and medical definitions being dependent on context, an interviewee said:

I think the line between a social trait and a medical trait is a socially constructed one that changes over time and that tends, in our society, to move according to how much people are aware of or how salient to them the role of biology is. And how much it’s something that society uses, in some way, as an object of blame or praise. I think we take things that we want to reward people for or punish people for and we assign them the category “social.” And that things that we want to say people are not blamed for and that we think of as really biological, and we put them in the category of “disease.” And that those categories are themselves socially constructed and pretty fuzzy (P10).

Another interviewee similarly highlighted the role of society in making determinations for what is social and medical. Noting that while “crisp answers” describing what is social and what is medical are impossible, “it’s more of a majority rule case where you say, ‘Well, it’s more social than a typical trait’ or ‘It’s more health than a typical, than the spectrum of traits we’re looking at.’… There’s just so many examples where you would bring it up and just say, ‘Well, which one is this? Well, which one is this?’ And there’s probably few examples that most people would nominate as only a health trait or only a social trait” (P11). Together, these interviewees highlight how distinctions between medical and social traits are fluid, shaped by changes in diagnostic criteria and social values.

### Deconstructing the line

Some respondents emphasized the fuzziness of medical and social categories given the vast entanglement of social and medical factors and sought to reenvision the idea of a line distinguishing the two, at times entirely rejecting the idea of strict categories. For instance, one interviewee said that the categories of medical and social traits are “too binary… I like to think of things, which we typically call medical traits, which could be sort of biologically proximal, so things that there’s a really short chain between impacts of a given gene and then what it does, and then go to much more biologically distal traits, which are typically classed as our social traits” (P12). Others similarly thought it was more helpful to move away from a binary approach to instead think of social and medical traits as a “continuum rather than as a sharp distinction” (P13). But as one such interviewee said:

There are certainly phenotypes like blood pressure and cholesterol levels which are at the extreme end of that continuum as in medical traits, and other traits like criminality that are at the other extreme of being very social and behavioral. But I think, in general, most traits are going to be somewhere in between. And even for things like cholesterol, that’s related to what you eat, which is related to cultural factors and other behavioral factors, like self-control. I’m not sure we can define any pure types of either, in either direction (P13).

Another respondent similarly referenced cholesterol and highlighted the difficulty of identifying purely medical or social traits, saying, “cholesterol, things like that, may be more of a medical trait, but they definitely have [a] social component in it. And education…is related to a lot of health outcomes. So, education by itself may be considered both a social trait and somewhat a medical trait. So, it’s really hard…” They also identified subjective wellbeing and risk-taking as more social traits, but added that “if you’re linking risk-taking to impulsive behaviors, then it may not be a pure social trait at all. So, I think the distinction is quite hard” (P14).

Given the difficulty of drawing a line between social and medical traits, some interviewees rejected the idea of drawing a line between the two altogether. For instance, when asked how they would distinguish social and medical traits, one said, “I can’t say I’ve been asked that question before. I’m not immediately convinced that that’s a reasonable question. So, the amount of time I spend exercising, is that a social trait or a medical trait?” (P15). Similarly, an interviewee immediately responded with a question, asking, “Why is this distinction so important?” (P16). They continued, saying, “I don’t really see a clear distinction. A lot of medical outcomes have a lot to do with behavior and lifestyle which can be partly social. I think they are quite intertwined. You can put it into categories…, but they are also really correlated. I don’t think it makes a lot of sense to really strive to make a really clear distinction here. And I don’t really see the merit” (P16). In sum, these respondents focused on the intertwining of social and biological factors in traits, with some actively rejecting any attempts to draw a clear line between social and medical.

### The necessity of the line

Across these varied reflections, some respondents considered whether and when drawing a line between social and medical traits might be necessary. For example, one interviewee described the need for the social/medical line for policy, saying:

I think that gives us something concrete to talk about in terms of how we prioritize our focus and how we even make decisions about government policy and government resources and public safety. So if we classify something as, say, a health outcome, that might suggest a kind of a whole different routine and maybe even puts it in that camp of ‘this is something that needs to be treated that we assign certain resources to,’ that if it’s to a social outcome, maybe it’s a different set of resources, a different level of priority (P17).

Another echoed this sense of necessity for governance, even while acknowledging challenges of using the line. They said that:

It’s quite important to draw that line because something should be controlled in regulation, like medical phenotypes could be, it should be, if it’s gonna be in the healthcare system, it should be regulated. But other than that, the other social traits, I’m not sure how that could be regulated because it’s outside of the system… So that could be an issue. So we might need a clear distinction between medical and non-medical (P2).

In several cases, respondents who questioned the validity of the distinction still, somewhat grudgingly, acknowledged its potential necessity for governance and policy purposes. One such interviewee said, “I think it’s a trap to draw a line,” but said:

You may draw a line for funding, accountancy purposes that, you know, somewhere the buck has to stop here, literally and figuratively. And I appreciate that. Just like a decent diagnosis…, at some point, you have to put a threshold and say if you fall to the right of this threshold, you will have a diagnosed disorder and then we could allocate the funds to treat that. I get that. But in reality, the reality is that life’s more of a continuum” (P18).

Similarly highlighting that even a flawed boundary may have necessary uses, another interviewee commented, “[I] don’t think there’s a magic point above which you are categorized as having a disorder and then one below which you definitely don’t. I just don’t think there’s a line, but insurance and clinical provision needs that to be a line” (P19).

Given the flawed boundary, however, some interviewees advised steering clear of drawing the line altogether when overseeing PGS research, indicating that the line may be unnecessary. When asked how they would distinguish the two, one said:

I think that’s difficult. I think rather than trying to draw some line somewhere, I think it should just be a consideration before people start this research of is there something beneficial for people’s health that could emerge from doing this analysis rather than just people saying, ‘Oh, this should be interesting certainly, and we get a paper out of it. So let’s just do it.’ There should be kind of more, maybe, internal review boards and things that say, ‘Before we do a study on polygenic risk scores, we should vet whether this is gonna be potentially beneficial or negative to people’s health’ (P20).

While this interviewee valued PGS for traits that can contribute to health, other interviewees worried about potential assumptions that PGS for traits related to health have more inherent value. As one interviewee argued, drawing the line between social and medical traits for policy purposes may work to absolve researchers of responsibility for the implications of their work if they fall on the medical side of the line. They said:

I think that one thing that falls out of these kind of conversations on “should we be looking at social traits” is this implication that, therefore, all medical traits, anything that can be classed as medical/clinical, is de facto fine. And I think that’s a quite dangerous position to make or to take because just because something is more biologically proximal, it does not negate the need to think very carefully about what’s being measured and the potential uses of it… (P12).

## Discussion

Our findings from interviews with scientists using PGS in their research highlight the difficulty of distinguishing between social and medical traits. Overall, interviewees broadly agreed that the line is ambiguous; they did not hold unequivocal views about what constitutes a social versus a medical trait, nor where a clear line might be drawn. Instead, their perspectives revealed a range of tensions. Some respondents reasoned through the distinction by suggesting that medical traits are more easily discerned than social ones while also acknowledging that the two are often deeply intertwined. Some emphasized that the overlap between social and medical traits makes any attempt at line-drawing meaningless. Yet, at times they reflected that, in practice, such lines are already being drawn for policy and governance purposes. These varied ways of thinking about the social/medical divide underscore key considerations for researchers and policymakers as they navigate the development and application of PGS across a broad spectrum of traits.

In the face of ambiguity around the distinction between social and medical traits, respondents at times relied on a biomedical model to define what counts as “medical,” pointing to clinical criteria, treatments, and diagnoses as markers. Yet these attempts to draw boundaries around what is medical were just that—attempts, underscoring the depth of conceptual ambiguity surrounding these categories. None of the interviewees articulated a clear or consistent criterion for distinguishing social from medical traits. Instead, many respondents noted that potential “medical” criteria are also fluid and shaped by social context, revealing uncertainty about whether such a line can be meaningfully drawn. This lack of clarity is not unique to PGS research but reflects longstanding debates about how health and disease, or pathology and normal variation, are distinguished. Discourse spanning medical sociology, medical anthropology, philosophy of science, and science and technology studies has addressed how health and pathology concepts are more “fuzzy” than fixed and that attempts to draw a line are often rooted in social processes, including normative judgements, rather than purely objective criteria (Sadegh-Zadeh 2000; Hesslow 1993; Canguilhem 1978). Thresholds for treatment or diagnosis, definitions of diseases, and determinations of what qualifies as a disease or medical condition vary across time and place, reflecting the process of medicalization by which aspects of everyday life come under the authority of medical science and providers (Conrad 2007). Relying on medicalized concepts to delineate medical from social traits may inadvertently reinforce or expand the scope of what is considered “medical,” a concern some interviewees raised, particularly in relation to neurodiversity.

Expanding what falls within or outside the purview of medicine raises ethical concerns for research using polygenic scores. For instance, some interviewees worried about assumptions that polygenic scores for medical traits aimed at improving health are inherently less ethically problematic than scores for social traits. Such assumptions may be linked to a perception that therapeutic or preventive goals are legitimate and socially desirable (Lantz et al. 2023), which may contribute to greater public acceptance of PGS research for medical traits compared to social traits (Long et al. 2025; Barlevy et al. 2024; Furrer et al. 2024; Zhang et al. 2021). Additionally, medical research and care are regulated in the U.S. through standards like CLIA for genetic testing labs, FDA oversight, IRB review, and even NIH funding requirements (Wolf et al. 2020). While such oversight is not without gaps, it may contribute to impressions that research on medical traits operates within stronger guardrails, reinforcing the sense that it is ethically safer, even though significant risks remain. While some scholarship has begun to address ethical concerns related to medical PGS (Trinidad et al. 2025; Lewis and Green 2021; Polygenic Risk Score Task Force 2021), an assumption that research on medical traits carries fewer concerns risks obscuring the social and ethical responsibilities of PGS researchers and the scientific limitations of their work, simply because their work is categorized as “medical.” Moreover, as the boundary between social and medical traits continues to shift through processes of medicalization, more research may be viewed as ethically acceptable solely because it falls on the “medical” side of the line. As a result, framing any polygenic score research as “just for health” may serve to not only distance researchers from a more critical engagement with the broader implications of their work, but could also mask the social and ethical consequences of expanding medical authority into areas of human life that are deeply contextual and contested (Kaczmarek 2019; Parens 2013).

Such ethical ambiguity also has implications for governance as law and policy regularly rely on a dividing line between these two concepts, even when not necessarily defining the line (Callier and Prince 2024; Kasperbauer and Wright 2020; Spector-Bagdady et al. 2018). Thus, depending on how a trait is viewed, the governance structures applying to PGS research or utilization could also be quite varied. As a hypothetical example, a researcher interested in developing a polygenic score for risk tolerance would first need to gain access to genetic data from a biobank. Access to such data is contingent on the informed consent provided by participants, which sometimes specifies whether future research would be medically related. As the UK biobank consent states, “I give permission for access to my medical and other health-related records, and for long-term storage and use of this and other information about me, for health-related research purposes (even after my incapacity or death)” (UK Biobank Consent 2025). However, even if biobanks attempt to restrict research to medical or health-related traits, such boundaries are inconsistently drawn, may be difficult to enforce, and may not align with how biobank participants interpret consent language (Meyer et al. 2023; Martschenko et al. 2025). Relatedly, if seeking funding, the researcher may need to emphasize the medical or health implications of their inquiry since the mission of many funding agencies is health focused (National Institutes of Health 2025). Thus, this researcher would want to emphasize the medical aspects of risk tolerance in order to secure funding and data sources. Over time, the researcher may develop a PGS for risk tolerance that attracts the attention of the public, potentially leading a direct-to-consumer company (DTC) to become interested in testing consumers and returning risk tolerance results. Whether such use by the DTC is regulated by the U.S. FDA depends on whether it is medical or social as the FDA has purview over medical tests but does not regulate apps and DTC tests for general wellness (Callier and Prince 2024). Similarly, whether state-level regulation and anti-discrimination protections apply to this DTC result may also depend on whether risk-tolerance is conceptualized as more medical or more social. Many state laws that regulate the collection, use, and privacy protections of genetic information define genetic test results as variants associated with increased risk of illness or disease (Spector-Bagdady et al. 2018). Accordingly, if law and policy establish risk tolerance as a non-medical trait, this would limit regulatory oversight and legal protections. As a polygenic score moves from research and development to use in society, different actors may be motivated to push for one interpretation or another depending on their underlying preferences for or against governance or regulatory oversight. PGS that sit in the blurry middle between medical and social are especially susceptible to this potential strategic framing.

Given the ethical and governance implications of the ambiguous boundary between medical and social traits, there may be reason to explore alternative frameworks for regulating PGS research and its applications. Such an alternative approach aligns with some interviewees’ attempts to deconstruct the social/medical distinction or reject it altogether, which may reflect the desire to move beyond dichotomies like nature/nurture, pathology/normal variation, or therapy/enhancement described by some as mired in unproductive false dichotomies or even “conceptual straightjackets” (Hesslow 1993:1; Sadegh-Zadeh 2000; Levitt 2013). The persistence of the therapy/enhancement debate in gene editing illustrates how challenging these boundaries does not automatically yield clear regulatory guidance as governance frameworks remain anchored in these categories that science and ethics increasingly contest (Santos 2025; Waltz et al. 2024; Juengst 2021). Learning from this experience, regulation of PGS might benefit from models that do not depend on rigid binaries but instead accommodate gradations of purpose, context, and social meaning (Lucivero et al. 2015). However, attempts to deconstruct or reject the social/medical distinction may also reflect the idea that policy questions around any potential boundaries of PGS use are premature, needing to be put off until more research provides clarity, a stance observed in other situations of scientific uncertainty (Campbell 1985; Murphy 2006; Tolwinski 2013). Yet given that PGS are currently marketed and commercialized by companies offering reproductive genetic testing services (Callier and Prince 2024), policy questions are both relevant and timely while the boundaries remain contested, including when determining permissibility of data access and use to generate PGS in the first place.

## Conclusion

This interview study reveals that the scientists most familiar with PGS methods and dissemination experience significant uncertainty about trait categorization. Such uncertainty, and even moral disagreement, is common among those experiencing emerging technologies or at the advent of analytic methods (Li et al. 2018; Nickel 2020; De Graeff et al. 2021; Nickel et al. 2022). Yet importantly, participants questioned whether drawing a boundary between medical and social traits is practical or desirable. Indeed, using such a boundary as a basis for PGS regulation could work to shield researchers of accountability when their work is framed as medical, while leaving research on social traits without adequate oversight. These reflections suggest that regulatory approaches premised on a clear separation between medical and social domains are ill-suited for the complexities of PGS research, calling for governance frameworks that address ethical risks across the full range of traits rather than relying on a line that many see as unstable or unworkable.

## Data Availability

The participants of this study did not give consent for their data to be shared publicly, so supporting data is not available.
